# Translation and validation of the anticipated turnover scale for the Portuguese cultural context

**DOI:** 10.1002/nop2.521

**Published:** 2020-06-10

**Authors:** Susana Isabel Rodrigues de Sul, Pedro Ricardo Martins Bernardes Lucas

**Affiliations:** ^1^ Nursing Research and Development Unit (UI&DE) Lisbon Nursing School Lisbon Portugal; ^2^ Central Lisbon University Hospital Centre Lisbon Portugal

**Keywords:** management, nursing, turnover, validation studies, work environment

## Abstract

**Aim:**

This study aimed to culturally and linguistically adapt the Anticipated Turnover Scale (ATS) for the Portuguese population.

**Design:**

A cross‐sectional study.

**Methods:**

The ATS instrument was validated in a non‐probabilistic sample of 259 nurses working at three hospitals in Portugal. The validity was assessed through factor analysis and structural equation modelling. Reliability was assessed using Cronbach's alpha, composite reliability and test–retest.

**Results:**

The final scale is a one‐factor scale consisting of 10 items and called "turnover intention." There are significant correlations among the scale items. Reliability and validity are acceptable. The ATS—Portuguese version, exhibited good psychometric properties for the Portuguese population.

## 1 INTRODUCTION

In recent years, the strong possibility of a short‐term shortage of nurses worldwide has been an issue of growing concern; the main reasons for the potential shortage are the ageing of the population and the working conditions of these professionals, which are still not optimal (Organization for Economic Co‐operation and Development, 2017). According to the World Health Organization (2013) and the International Council of Nursing (ICN) (2018), it is estimated that there will be a global deficit of approximately nine million nurses by 2030.

The phenomenon of emigration among health professionals, especially nurses, that has been observed in recent years also calls into question the equitable distribution and sustainability of health services, which is why the report of the Organization for Economic Co‐operation and Development (OECD) & European Observatory on Health Systems and Policies (EOHSP) (2017) highlights the extreme need to be able to motivate professionals and contain and reverse their intention to leave the country.

The study of the turnover phenomenon should begin with the study of turnover intention; that is, studying turnover intention is important for identifying and anticipating turnover and may ultimately enable it to be avoided. Several studies have shown a strong positive relationship between the intention to leave a job and actually doing so, which is why the study of turnover should focus on both facets (Galleta, Portoghese, Carta, D'Aloja, & Campagna, 2016; Hinshaw & Atwood, 1984).

It is important to note that the turnover phenomenon accommodates the possible entry of new elements that are motivated by and promote medium‐term innovation in the department that employs them. However, turnover has the potential for a great negative impact (Li & Jones, 2012) because this departure process is associated with negative nursing practice environments, a large loss of intellectual capital and threats to the safety of the staff and the safety and quality of care; it can lead to increased hospital stays, loss of users to other health institutions due to lack of resources and an increased occurrence of incidents (Chênevert, Jourdain, & Vandenberghe, 2016; Fallatah, Laschinger, & Read, 2017).

Turnover it is also an expensive process as it requires the recruitment, hiring and integration of new nurses, which jeopardizes the sustainability of health services (Hunt, 2009; Kovner, Brewer, Fatehi, & Jun, 2014). According to the study by Colosi (2016), the cost of the turnover of a nurse in a hospital setting in the United States ranges from $37,700–$58,400, representing a tremendous challenge for healthcare organizations in the search for profit and sustainability (Colosi, 2016; Li & Jones, 2012).

The departure of nurses from a given nursing environment represents an overload for colleagues who remain, and these colleagues tend to be less satisfied, leading to a decrease in the quality of relationships and in team cohesion (Fallatah et al., 2017). Nevertheless, according to Kutney‐Lee, Wu, Sloane, and Aiken (2013), nursing practice environments are mutable, that is the intervention of the nurse manager can alter the environment and, ultimately, improve it.

Thus, providing nurse managers with a scale that allows them to evaluate nurses' intention to abandon their current job is important so that nurse managers can make an initial diagnosis of their team's situation (Lake, 2007). Given that in Portugal, there is no scale for evaluating turnover intention in nurses specifically, and this study aims to translate, validate and culturally adapt a scale that evaluates such intention for the Portuguese population for future use by nurse managers or researchers.

## 2 METHODS

### 2.1 Design

The present study is a scientific quantitative, observational and descriptive cross‐sectional study. The objective of the present study was to culturally and linguistically adapt the Anticipated Turnover Scale (ATS) of Hinshaw and Atwood (1984).

### 2.2 Study type, participants and data collection

A self‐administrated questionnaire survey was conducted on Registered Nurses in 3 hospitals in the Lisbon area, in Portugal. The hospitals included were public and university hospitals. All nurses from the three hospitals were invited to participate in the study and were approached after prior meetings with the head nurses of each department. Those who agreed to participate in the study were given envelopes containing a demographic form (that did not collect personally identifiable information), the scale and instructions on how to complete the scale.

Data were collected between December 2018–January 2019. Among the 550 distributed questionnaires, 297 were returned and 259 included valid responses satisfying both inclusion criteria: participants should be working as Registered Nurses at the selected hospitals and not have missed providing answer for any variable.

### 2.3 Measurement development

#### 2.3.1 Original version of the anticipated turnover scale

The original data collection instrument is called the Anticipated Turnover Scale (ATS). This scale, developed by Hinshaw and Atwood in 1984, evaluates the intention of nurses to voluntarily abandon their current job, either through internal or external turnover or abandoning the nursing career. The ATS consists of 12 items scored on a Likert‐type scale ranging from 1 (strongly disagree)–7 (strongly Agree). It includes six reverse‐scored items to avoid bias. The total variance of the results may range from 12–84 points, and the higher the score, the higher the risk of turnover is (Barlow & Zangaro, 2010; Hinshaw & Atwood, 1984).

The scale continues to be widely used in its country of origin, the United States of America (Brady‐Schwartz, 2005; Hart, 2005; Hunt, 2013; Shader, Broome, Broome, West, & Nagle, 2001; Volk & Lucas, 1991), and is considered easy to complete and of an appropriate reading and comprehension level for nurses. It is expected to take approximately 5 min to complete. Scoring and interpreting the results are also considered simple (Barlow & Zangaro, 2010).

Regarding its validity and reliability, in the original study by Hinshaw and Atwood (1984), the internal consistency according to Cronbach's alpha was 0.84 and the factor analysis revealed two factors that explained 54.9% of the variance.

#### 2.3.2 Translation and cultural adaptation—content validity

Using the methodology proposed by Beaton, Bombardier, Guillemin, and Ferraz (2000) and Sousa and Rojjanasrirat (2011), the scale was translated from English into Portuguese by two independent translators, one with knowledge in the nursing field. An analysis of the two versions was performed and a consensus version was generated.

The consensus version was then back translated by a third translator. At this step, it was observed that all 12 statements translated back into English maintained the same meaning as the original statements.

Finally, a comprehensive pretest of the new Portuguese version of the ATS was piloted on a non‐probabilistic sample of nurses. If more than 20% of the individuals had doubts about the scale, it would have to be re‐analysed and possibly re‐translated (Beaton et al., 2000; Sousa & Rojjanasrirat, 2011). The scale was completed by 22 nurses (non‐probabilistic sample). Approximately 86% of this sample did not encounter any problems with the scale during its completion. They considered it a quick and simple questionnaire and understood the meaning of all the statements presented. The remaining 14% of the sample posed a very pertinent question regarding the similarity of two items (1 and 10). This issue was clarified by reviewing the adaptation and validation process with the author, Dr Atwood, to discuss the difference between the two items and then seeking to more clearly differentiate them.

### 2.4 Statistical data analysis

The data were analysed using the software IBM^®^ SPSS^®^ Statistics version 25.0 and AMOS (v.21, SPSS, Inc.).

#### 2.4.1 Reliability

The reliability of the instrument was determined using Cronbach's alpha coefficient and the composite reliability. These values can range from 0–1, and good internal consistency should exceed an alpha of 0.80; however, in newly created scales or scales with a low number of items, values > 0.60 are acceptable (Grove, Sutherland & Grey, 2017; Marôco, 2014). Composite reliability was considered adequate when it was equal to or greater than 0.70 (Marôco, 2014). The temporal stability of the instrument, that is its reproducibility, was also analysed using the Pearson correlation for paired samples with a normal distribution. These values range from zero–one and the closer to one the value is, the greater the instrument's temporal stability (Grove, Sutherland & Grey, 2017).

#### 2.4.2 Validity

Content validity was ensured throughout the translation and adaptation of the scale for the Portuguese population with the help of professionals and the validation test (pretest) (Grove et al., 2017). The structural validity of the scale was tested first using exploratory factor analysis and then through structural equation modelling and confirmatory factor analysis (CFA) by means of factorial validity and convergent validity. The factorial validity was tested with maximum likelihood estimation. The proposed factor model was considered valid when all items presented factor weights greater than 0.4 (Almeida, 2017). The convergent validity was estimated by the average variance extracted (AVE) (Marôco, 2014).

## 3 ETHICAL CONSIDERATION

Authorization to translate and validate the original version of the scale was requested from the scale's author. Authorization to conduct the present study was requested from and granted by the Board of Directors and the Ethics Committee of the selected hospitals. The study respected the principles of autonomy and the right to confidentiality and anonymity by obtaining informed consent.

## 4 RESULTS

### 4.1 Description of the sample

The Portuguese version of the ATS was administered to 297 Registered Nurses (RNs) to assess the scale's psychometric properties, and the response rate was 59.4%. However, 38 questionnaires were excluded because they were not completed. The final sample consisted of 259 nurses aged between 22–60 years, with a mean age of 36 (35.8) (Table 1). According to the Instituto Nacional de Estatística (2018), the Order of Nurses indicated that in 2016, 68.8% of working nurses were between 31–60 years old. Regarding the study sample, 66.4% of the nurses were between 31–60 years of age; most (78.4% of all respondents) were female. The results relative to gender agrees with the Portuguese nursing reality.

**Table 1:** Demographic characteristics

Demographic details	Frequency (*N*)	Percentage
Gender		
Female	203	78.4
Male	56	21.6
Position		
Nurse	249	96.1
Nurse specialist	9	3.5
Nurse manager/chief	1	0.4
Level of nursing education		
Bachelor degree	233	90.0
Master degree	24	9.2
Doctorate degree	2	0.8
Place of work		
Impatient departments	163	62.9
Intensive care units	57	22.0
Emergency services	16	6.2
Operating rooms	15	5.8
Outpatient clinics	8	3.1
Employment status		
Permanent ccontracts	177	68.3
Temporary contracts	4	1.5
Public sector employment contracts	78	30.1

	Mean
Age	35.8
Years of nursing experience	12.6
Years of working in the same place of work	11.1

Bold values are the only ones superior to the Cronbach's alpha for the 12 items of the scale (.865).

### 4.2 Reliability and validity

Based on the twelve items of the scale, the reliability of the scale was analysed and internal consistency was determined using Cronbach's alpha which was 0.865. Table 2 shows the correlation between all items and the total for the scale, as well as the behaviour of Cronbach's alpha when an item was excluded.

**Table 2:** Reliability (Cronbach's alpha) coefficient values for each item

	Corrected total item correlation	Cronbach's alpha if the item was excluded
Item 1—I intend to stay in my current job for some time.	.800	.839
Item 2—I am almost certain that I will leave my job in the near future.	.694	.845
Item 3—Deciding whether to stay or leave my job is not an essential issue for me at this time.	.412	.864
Item 4—I have already made the decision to stay with or leave this organization in the short term.	.232	**.878**
Item 5—If I were to receive another job offer tomorrow, I would seriously consider it.	.548	.855
Item 6—I have no intention of leaving my current job.	.790	.837
Item 7—I have been at this workplace for as long as I want to be.	.498	.858
Item 8—I am sure that I will stay here for a while.	.746	.844
Item 9—I have no specific idea how much longer I will stay here.	.135	**.893**
Item 10—I intend to keep my job at this organization for some time.	.803	.841
Item 11—I have major doubts about whether or not I will stay in this organization.	.549	.855
Item 12—I plan to leave this job soon.	.844	.836

Bold values are the only ones superior to the Cronbach's alpha for the 12 items of the scale (.865).

To determine whether the data collected were adequate for the exploratory factor analysis, the Kaiser–Meyer–Olkin (KMO) and the p‐value of Bartlett's test of sphericity were determined*,* with values of 0.922 and <0.001, respectively, which are considered excellent for the use of factor analysis (Grove et al., 2017). With an eigenvalue of 1 and the scree plot criterion, it was observed that a 2‐factor matrix explained 58.89% of the total variance. The first component consisted of items 1, 2, 3, 5, 6, 7, 8, 10, 11 and 12, and the second component comprised items 4 and 9 (Table 2). After the factors were extracted, the extent to which the variables saturated these factors was calculated through varimax orthogonal rotation and a cut‐off value of 0.4 was adopted, as recommended by Almeida (2017) (Table 3).

**Table 3:** Factor analysis of the 12 items with eigenvalue of 1

	Rotation matrix component	
	1	2
Item 1	**0.858**	0.111
Item 2	**0.774**	0.085
Item 3	**0.477**	0.196
Item 4	0.219	**0.629**
Item 5	**0.657**	−0.132
Item 6	**0.872**	−0.029
Item 7	**0.614**	−0.178
Item 8	**0.834**	0.032
Item 9	−0.211	**0.808**
Item 10	**0.861**	0.111
Item 11	**0.646**	−0.002
Item 12	**0.899**	0.100

Bold values are the only ones superior to the Cronbach's alpha for the 12 items of the scale (.865).

Next, Cronbach's alpha was calculated for subscale 2, consisting of items 4 and 9, and the result was 0.219. Item 9 had a correlation coefficient of 0.135, so it was excluded. Calculating Cronbach's alpha again for the 11 remaining items showed that item 4 had a correlation coefficient of 0.197, and thus, it was also excluded (Table 4).

**Table 4:** Factor analysis for the 11 items with an eigenvalue of 1

	Corrected total item correlation	Cronbach's alpha if the item is excluded
Item 1	.821	.875
Item 2	.713	.880
Item 3	.389	.901
Item 4	**.197**	.914
Item 5	.558	.890
Item 6	.796	.874
Item 7	.524	.891
Item 8	.834	.876
Item 10	.835	.876
Item 11	.582	.888
Item 12	.863	.872

Bold values are the only ones superior to the Cronbach's alpha for the 12 items of the scale (.865).

Then, the analysis of the structural validity of the scale was repeated without the two excluded items (4 and 9). KMO and Bartlett's test of sphericity results were 0.934 and <0.001, respectively, which are once again considered excellent for the use of factor analysis. With an eigenvalue of 1 and the scree plot criterion, it was observed that a matrix consisting of one (1) factor explaining 58.18% of the total variance. As only one component was extracted, the solution did not undergo rotation. The reliability of the scale was then evaluated again by analysing the internal consistency, and a Cronbach alpha of 0.91 was obtained, which corresponds to a very good internal consistency. Table 5 shows the correlation between the remaining items and the total scale.

**Table 5:** Reliability (Cronbach's alpha) coefficient values for the 10 items

	Corrected total item correlation	Cronbach's alpha if the item is excluded
Item 1	.811	.893
Item 2	.703	.899
Item 3	**.412**	**.919**
Item 5	.568	.908
Item 6	.824	.891
Item 7	.526	.910
Item 8	.769	.896
Item 10	.816	.894
Item 11	.567	.907
Item 12	.853	.890

For reliability, temporal stability was also tested using the test–retest method. The instrument was administered to 22 nurses who completed the questionnaire twice with an interval of 2 weeks, during which the participants reported that their turnover intentions had not significantly changed. By analysing the Pearson correlation coefficient *r* and excluding items 4 and 9, a value of .774 was obtained, which is considered a strong correlation. The scale was thus considered reliable.

#### 4.2.1 Confirmatory factor analysis

A preliminary CFA was performed for the one‐factor solution with 10 observable variables using the maximum likelihood method. The analysis revealed that no variable showed values of asymmetry and kurtosis indicating severe violations of the normal distribution (|Sk|<3 and |Ku|<10) (Marôco, 2014); thus, the instrument presented an acceptable multivariate kurtosis value (KuMult = 46,786) (KuMult < 70 deviation from normality non‐critical for CFA). The original model was fitted to a sample of 259 individuals and showed a satisfactory fit (*χ* ^2^_(35)_ = 103,286; CMIN/DF = 2.951; GFI = 0.902; CFI = 0.783; PCFI = 0.609; RMSEA = 0.087; P[rmsea ≤ 0.05] = 0.001). To improve the fit of the global model, 12 observations whose D^2^ values suggested that they were multivariate outliers (p1 and p2 < 0.001) were excluded from the CFA. As suggested by the modification index, a path between the residuals of variables B_P3 and B_P5 was included in the model. As a result, the multivariate kurtosis value decreased by approximately half (KuMult = 25.157) and the goodness‐of‐fit was satisfactory to good, with an improvement in the respective indices (*χ* ^2^_(34)_ = 81.529; CMIN/DF = 2.398; GFI=0.926; CFI=0.841; PCFI=0.635; RMSEA=0.075; P[rmsea ≤ 0.05] = 0.024), thus showing that the model was adequate.

**FIGURE 1:**
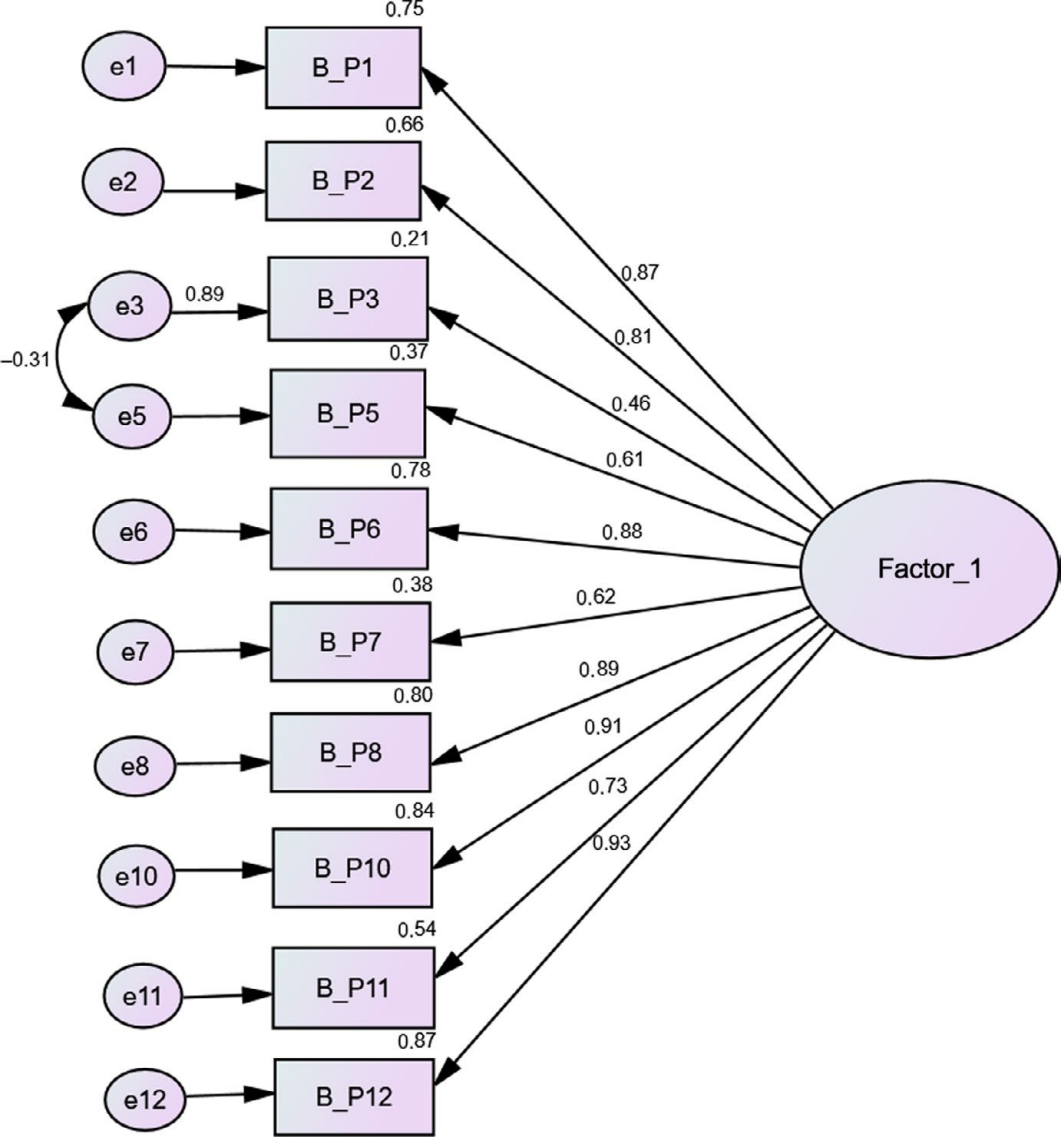
Confirmatory factor analysis for validating the scale items

Figure 1 shows the model values in terms of local fit, namely the standardized factor weights and the individual reliability of each item. The composite reliability of the factor was high, with a value greater than 0.7 (CR = 0.940) and appropriate convergent validity, with a value greater than 0.5 (AVE = 0.619). The common factor (factor 1) has excellent reliability index (Cronbach's alpha = 0.910); therefore, the solution obtained was considered viable for subsequent analyses.

## 5 DISCUSSION

### 5.1 Reliability and Validity

As the results show, the reliability of the scale was improved by the presence of all items except for items 9 and 4, which reduced the reliability of the scale. However, those items were retained in a first moment so that their behaviour could be analysed during the factor analysis. Later, the items were excluded because according to Streiner and Norman (2008), items must have a correlation higher than 0.200 with the total scale. Item 3 shows a correlation with the total scale score that was lower than that of the remaining items (0.412), slightly lowering Cronbach's alpha of the scale total. However, because this was the first time that this scale was being used in the Portuguese cultural context, we decided to keep the rest of the scale unchanged. The modification suggestions of the software IBM®SPSS® Amos v.21.0.0, which was used to conduct the CFA, suggested positioning item 3 as covariate with item 5. The covariance of this pair may be associated with the existence of a source of common variation of the items not fully explained by the common factor in the model (Marôco, 2014).

### 5.2 Comparison of the original scale with the Portuguese version

Regarding the results, the exploratory factor analysis differed from the original study, which obtained two factors that explained 54.9% of the variance (Hinshaw & Atwood, 1984). Nevertheless, according to Barlow and Zangaro (2010), Hinshaw noted that the ATS had no subscales, making it a one‐factor instrument. Thus and taking into account the results obtained, it was considered that the extracted factor corresponded to the turnover intention, which was the only structural that the scale evaluated. All ten (10) items contribute to explaining this phenomenon.

### 5.3 Anticipated turnover scale—Portuguese version

With the scale reduced to 10 items, it is worth noting that the maximum score will be 70 points and the minimum will be 10 points and the higher the score is, the higher the turnover intention. It should be noted that in the translated version, the items that are negative and reverse scored are items 1, 3, 5, 7 and 8. The Portuguese version of the ATS is thus called “*Escala de Intenção de Turnover.”* The scale evaluates the turnover intention; however, its results do not allow for any hypothesis regarding what promotes turnover intention to be drawn. It is also recommended to always provide, when applying this scale, a brief explanation regarding what is considered a turnover intention because the intention may be to abandon the present position (department/unit), the organization or even the profession.

### 5.4 Implications for nursing

The present study aimed to contribute to combating turnover by providing a method that would help with the early assessment of this intention in the Portuguese cultural context to avoid such a phenomenon. We understand that the research conducted in this study is relevant for nursing management and offers important contributions not only for future research but also for nursing practice, namely for nursing team management. This is why we hope that this instrument will be widely used, both in nursing practice and in scientific research. The present scale provides a substantial contribution to nursing and contributes to the improvement of the nursing practice environment in the various nursing work settings, including hospitals, primary health care facilities, integrated continuous care facilities or long‐term care facilities for elderly people.

### 5.5 Limitations and future research

This study has some limitations. First the criterion validity was not assessed as there is no other scale that evaluates this same aspect—turnover intention—in the Portuguese population. Thus, it was not possible to evaluate the sensitivity of the ATS against another scale that evaluates the same aspect. Second, the targeted people of this study were nurses working in hospital wards, so future research should confirm the applicability of the scale in various settings.

## 6 CONCLUSION

The adopted methodology and the data obtained from the sample are acceptable for the purpose of the present study; the conviction remains that the methodological steps were rigorously executed and based on scientific evidence. The CFA confirmed the one‐factor structure of the instrument. The ATS, after having undergone the necessary adaptations, presented good psychometric properties for the Portuguese population and proved to be a reliable and valid instrument for the evaluation of turnover intention; thus, we suggest that it be applied in future studies and in a broader way.

## CONFLICT OF INTEREST

The authors declare that there is no conflict of interest.

## ETHICAL APPROVAL

Name of the review board that approved the present study: *Comissão de Ética para a Saúde*, Central Lisbon University Hospital Centre.

## References

[nop2521-bib-0001] International Council of Nurses (2018). World's nurses need a pay rise and better working conditions, concludes new report. Atas de International Council of Nurses Workforce Forums. Geneva, Switzerland: ICN; 2018.

[nop2521-bib-0002] Almeida, S. (2017). Estatística Aplicada à Investigação em Ciências da Saúde. Loures: Lusodidacta.

[nop2521-bib-0003] Barlow, K. , & Zangaro, G. (2010). Meta‐analysis of the reliability and validity of the Anticipated Turnover Scale across studies of registered nurses in the United States. Journal of Nursing Management, 18, 862–873. 10.1111/j.1365-2834.2010.01171.x 20946222

[nop2521-bib-0004] Beaton, D. , Bombardier, C. , Guillemin, F. , & Ferraz, M. (2000). Guidelines for the process of cross‐cultural adaptation of self‐report measures. Spine, 25(24), 3186–3191. 10.1097/00007632-200012150-00014 11124735

[nop2521-bib-0005] Brady‐Schwartz, D. (2005). Further evidence on the magnet program recognition. Journal of Nursing Administration, 35(9), 397–403. 16200007 10.1097/00005110-200509000-00009

[nop2521-bib-0006] Chênevert, D. , Jourdain, G. , & Vandenberghe, C. (2016). The role of high‐ involvement work practices and professional self‐image in nursing recruits' turnover: A three‐year prospective study. International Journal of Nursing Studies, 53, 73–84. 10.1016/j.ijnurstu.2015.09.005 26421911

[nop2521-bib-0007] Colosi, B. (2016). National Healthcare Retention & RN Staffing Report. Pennsylvania: Nursing Solutions Inc.

[nop2521-bib-0008] Fallatah, F. , Laschinger, H. , & Read, E. (2017). The effects of authentic leadership, organizational identification and occupational coping self‐efficacy on new graduate nurses' job turnover intentions in Canada. Nursing Outlook, 65(2), 172–183. 28126250 10.1016/j.outlook.2016.11.020

[nop2521-bib-0009] Galleta, M. , Portoghese, I. , Carta, M. , D'Aloja, E. , & Campagna, M. (2016). The effect of nurse‐physician collaboration on job satisfaction, team commitment and turnover intention in nurses. Research in Nursing & Health, 39(5), 375–385. 10.1002/nur.21733 27233052

[nop2521-bib-0010] Grove, S. , Sutherland, S. , & Gray, J. (2017). The practice of nursing research: Appraisal, synthesis and generation of evidence (8th ed.). Missouri: Elsevier.

[nop2521-bib-0011] Hart, S. (2005). Hospital ethical climates and registered nurses' turnover intentions. Journal of Nursing Scholarship, 37(2), 173–177. 10.1111/j.1547-5069.2005.00030.x 15960062

[nop2521-bib-0012] Hinshaw, A. , & Atwood, J. (1984). Instrument: Anticipated turnover scale (ATS) from J. Atwood; 2018. [email] (personal communication, 23 September 2018).

[nop2521-bib-0013] Hunt, D. (2013). Does value congruence between nurses and supervisors effect job satisfaction and turnover? Journal of Nursing Management, 22(5), 572–582. 23829178 10.1111/jonm.12055

[nop2521-bib-0014] Hunt, S. (2009). Nursing turnover: Costs, causes & solutions. California: SuccessFactors Inc.

[nop2521-bib-0015] Instituto Nacional de Estatística (2018). Estatísticas da Saúde 2016. Lisboa: INE.

[nop2521-bib-0016] Kovner, C. , Brewer, C. , Fatehi, F. , & Jun, J. (2014). What does nurse turnover rate mean and what is the rate? Policy, Politics & Nursing Practice, 15, 64–71. 10.1177/1527154414547953 25156041

[nop2521-bib-0017] Kutney‐Lee, A. , Wu, E. , Sloane, D. , & Aiken, L. (2013). Changes in hospital nurse work environments and nurse job outcomes: An analysis of panel data. International Journal of Nursing Studies, 50(2), 195–201. 10.1016/j.ijnurstu.2012.07.014 22902135 PMC3589738

[nop2521-bib-0018] Lake, E. (2007). The nursing practice environment: Measurement and evidence. Medical Care Research and Review, 64(2), 104–122. 10.1177/1077558707299253 17406014

[nop2521-bib-0019] Li, Y. , & Jones, C. (2012). A literature review of nursing turnover costs. Journal of Nursing Management, 31(3), 405–418. 10.1111/j.1365-2834.2012.01411.x 23406301

[nop2521-bib-0020] Marôco, J. (2014). Análise de Equações Estruturais: Fundamentos teóricos, software & aplicações. Pêro Pinheiro: Report Number.

[nop2521-bib-0021] Organization for Economic Co‐operation and Development (OECD) & European Observatory on Health Systems and Policies ( EOHSP ) (2017). State of Health in the EU Portugal Perfil de Saúde do País 2017. Brussels: European Commission.

[nop2521-bib-0022] Organization for Economic Co‐operation and Development (OECD) (2017). Health at a Glance 2017: OECD Indicators. Paris: OECD Publishing.

[nop2521-bib-0023] Shader, K. , Broome, K. , Broome, C. , West, M. , & Nagle, M. (2001). Factors influencing job satisfaction and anticipated turnover in an academic medical center. Journal of Nursing Administration, 31(4), 210–217. 11324334 10.1097/00005110-200104000-00010

[nop2521-bib-0024] Sousa, V. , & Rojjanasrirat, W. (2011). Translation, adaptation and validation of instruments or scales for use in cross‐cultural health care research: A clear and user‐ friendly guideline. Journal of Evaluation in Clinical Practice, 17, 268–274. 10.1111/j.1365-2753.2010.01434.x 20874835

[nop2521-bib-0025] Streiner, D. , & Norman, G. (2008). Health and measurement scales. A practical guide for their development and use (4th ed.). Oxford, UK: Oxford University Press.

[nop2521-bib-0026] Volk, M. , & Lucas, M. (1991). Relationship of management style and anticipated *turnover* . Dimensions in Critical Care, 10(1), 35–40. 10.1097/00003465-199101000-00008 1989841

[nop2521-bib-0027] World Health Organization (2013). A universal truth: No health without a workforce. Geneva, Switzerland: WHO.

